# Association of Urinary Benzene Metabolite and the Ratio of Triglycerides to High-Density Lipoprotein Cholesterol: A Cross-Sectional Study Using the Korean National Environmental Health Survey (2018–2020)

**DOI:** 10.3390/toxics11120985

**Published:** 2023-12-04

**Authors:** Seungju Baek, Eunjung Park, Eun Young Park

**Affiliations:** 1 Department of Public Health, Korea University Graduate School, Seoul 02814, Republic of Korea; seungju100@korea.ac.kr; 2 Department of Cancer Control and Population Health, National Cancer Center, Graduate School of Cancer Science and Policy, Goyang 10408, Republic of Korea; eunjungpark@ncc.re.kr; 3 Department of Preventive Medicine, Korea University College of Medicine, Seoul 02814, Republic of Korea

**Keywords:** environmental exposure, benzene, toluene, TG/HDL-C ratio

## Abstract

The aim of this study was to investigate the association between benzene and toluene, and the ratio of triglycerides to high-density lipoprotein cholesterol (TG/HDL-C). This cross-sectional study analyzed 1928 adults using nationally representative data from the Korean National Environmental Health Survey (KoNEHS) Cycle 4 (2018–2020). Urinary trans, trans-muconic acid (t,t-MA) and benzylmercapturic acid (BMA) were measured by high-performance liquid chromatography–mass spectrometry (HPLC–MS/MS), and high-density lipoprotein cholesterol (HDL-C) and triglycerides (TGs) were analyzed by colorimetry. Survey logistic regression analysis was applied to examine the association between urinary t,t-MA and BMA and the TG/HDL-C ratio. Urinary t,t-MA is significantly associated with an elevated TG/HDL-C ratio in both men and women (for men, OR [95% (CI)]: 2nd quartile: 2.10 [1.04, 4.22]; 3rd quartile: 2.13 [0.98, 4.62]; 4th quartile: 2.39 [1.05, 5.45]; for women, OR [95% (CI)]: 2nd quartile: 1.21 [0.71, 2.06]; 3rd quartile: 1.65 [0.94, 2.90]; 4th quartile: 1.78 [1.01, 3.11]), with significant dose–response relationships (P for trend: for men, 0.029; women, 0.024). This study shows that environmental exposure to benzene is associated with the TG/HDL-C ratio in the Korean general population. This suggests that more stringent environmental health policies are needed to reduce benzene exposure.

## 1. Introduction

Benzene (CAS No. 71-43-2) is a colorless liquid classified as a Group 1 carcinogen by the International Agency for Research on Cancer (IARC) [1]. It is commonly encountered through environmental and occupational exposure, with major sources including cigarette smoke, gasoline exhaust, and industrial processes [2,3]. Benzene exposure primarily occurs through inhalation, with dermal contact and ingestion being less frequent routes of exposure [4]. Benzene exposure can result in both short-term and long-term effects on the human body. Acute exposure to high levels of benzene can lead to symptoms such as dizziness, fatigue, headaches, and, in severe cases, fatality. Chronic low-level exposure to benzene has been linked to neurological, hematological, and systemic effects, including impacts on the cardiovascular system [1,5,6]. 

Toluene (CAS No. 108-88-3) has similar chemical properties to benzene, as it belongs to the same group of Volatile Organic Compounds (VOCs). It is a clear liquid classified as noncarcinogenic to humans (Group-3) by the IARC [7,8]. Toluene occurs naturally and is released into the environment when it is used as a solvent in paints, adhesives rubber, and many other products, or in the production of other chemicals [7]. The main route of human exposure to toluene is through inhalation, and the symptoms of toluene exposure are mainly neurotoxic [7,9]. Chronic exposure to toluene can result in cognitive neurological deficits and can also affect renal, hepatic, and cardiovascular functions [7,9,10].

The growing evidence indicates an association between the ratio of triglycerides to high-density lipoprotein cholesterol (TG/HDL-C) and the risk of insulin resistance and cardiovascular disease [11,12,13]. According to previous studies, an elevated TG/HDL-C ratio plays an important role in identifying insulin resistance in children, adolescents, and adults [14,15,16]. A recent meta-analysis study demonstrated that the TG/HDL-C ratio is significantly associated with homeostatic model assessment index (HOMA-IR) levels, which is a simplified surrogate marker for quantifying insulin sensitivity [17,18]. The TG/HDL-C ratio is also an independent predictor of cardiovascular disease. Some previous studies have shown that high TG and low HDL-C concentrations are risk factors for ischemic heart disease (IHD). Decreased activity of lipoprotein lipase, a key enzyme in lipid metabolism, leads to elevated triglyceride levels. This results in increased remnants of chylomicrons and very-low-density lipoproteins (VLDL), while lowering HDL levels. These metabolic processes can contribute to the formation of atherosclerosis [19,20]. 

There is evidence suggesting that exposure to benzene with urinary trans, trans-muconic acid (t,t-MA) as a biomarker, might be significantly associated with the risk of insulin resistance as well as diabetes mellitus (DM) [21,22,23,24,25]. Previous mechanistic studies proposed that the production of oxidative stress during benzene metabolism plays a crucial role in the toxic effects of benzene. This oxidative stress has the potential to cause damage to DNA, proteins, and lipid peroxidation, which are potential pathophysiological factors contributing to various diseases such as diabetes, hypertension, and cardiovascular disease (CVD) [26,27,28,29,30,31]. However, there is limited epidemiologic evidence on the associations between exposure to benzene and toluene and blood lipid levels [32]. 

Therefore, the purpose of this study was to investigate the association between environmental exposure to benzene and the TG/HDL-C ratio (i.e., surrogate marker of insulin resistance, and cardiovascular disease). We conducted a cross-sectional study using urinary biomarkers (i.e., urinary t,t-MA for benzene and benzylmercapturic acid (BMA) for toluene), based on the Korean National Environmental Health Survey (KoNEHS) Cycle 4 (2018–2020). 

## 2. Materials and Methods

### 2.1. Study Population

The KoNEHS Cycle 4 (2018–2020), the nationally representative population, a two-stage, proportionally stratified sampling design, includes 4239 adults from the Korean population. The KoNEHS is a cross-sectional nationwide survey that has been conducted every three years since 2009. This survey provides basic data for national environmental health policies and contributes to health protection by investigating and analyzing the concentration of harmful substances in the body and their influencing factors. The blood and urine samples were collected from the representative Korean population. The survey was composed of obtaining detailed information regarding demographics, lifestyle, and environmental factors to explore the exposure routes, amount, and frequency of exposure in the environment through a structured questionnaire, clinical laboratory assessments, and analyses of environmental pollutants using blood and urine.

This survey was approved by the Institutional Review Board of the National Institute of Environmental Research (NIER), Korea (IRB No. NIER-2018-01-01-001) and was conducted only for individuals who provided prior consent. The reporting of this cross-sectional study followed the Strengthening the Reporting of Observational Studies in Epidemiology (STROBE) guidelines.

**Figure 1:**
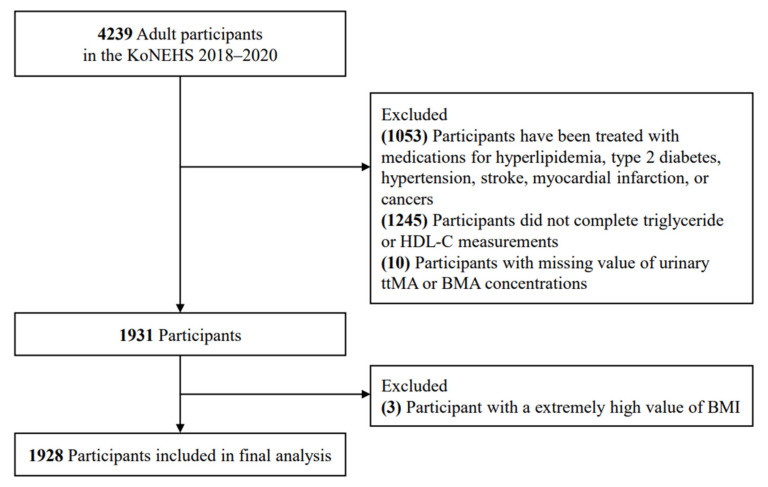
Flow diagram showing study sample derivation.

Among the 4239 adult participants aged over 19 years from the KoNEHS Cycle 4 (2018–2020), 1931 were eligible, after excluding 1053 participants who had been treated with medications for hyperlipidemia, type 2 diabetes, hypertension, stroke, myocardial infarction, or cancer, and 1245 participants who did not complete triglyceride or HDL-C measurements, as well as 10 participants with missing values of urinary t,t-MA and BMA concentrations. Additionally, 3 individuals with high values of body mass index (BMI) were further excluded. Finally, the data for 1928 participants (803 men and 1125 women) were included in the final analysis (Figure 1).

### 2.2. Case Ascertainment

According to the guidelines of the National Cholesterol Education Program Adult Treatment Panel III (NCEP-ATP III) and the American Heart Association (AHA), suspected cardiovascular disease was defined as NCEP-ATP III: TG/HDL-C > 3.75 in men and TG/HDL-C > 3 mg/dL in women; AHA: TG/HDL-C > 2.5 in men, >2 mg/dL in women.

### 2.3. Assessment of Serum Lipid Profiles 

The following serum lipid profiles were measured during the KoNEHS Cycle 4: HDL-C and TG. Whole blood samples were collected in either a plain tube, serum separation tubes (SST), or a trace element ethylenediaminetetraacetic acid (EDTA) tube. Blood samples were centrifuged at 3000 rpm to separate the serum and stored refrigerated (2~6 °C) [33]. HDL-C was measured by analyzing quinoneimine produced after a peroxidase reaction of H_2_O_2_ produced by cholesterol oxidase with a colorimetric analysis at 596 nm wavelengths (elimination/catalase method, Siemens ADIVA 1800, Erlangen, Germany) [34]. TG levels were measured by analyzing hydrolyzed glycerol produced by lipoprotein lipase (LPL) with a colorimetric analysis at 505/694 nm wavelengths (GPO Trinder without serum blank method, ADIVA 1800, Siemens) [34].

### 2.4. Measurement of Urinary Metabolites

The following urinary metabolites, t,t-MA and BMA, were measured according to the KoNEHS guidelines. Urine samples were collected in specimen cups and delivered in a refrigerated state (2~6 °C) to light-blocked storage containers. The urine samples were kept frozen at −20 °C before analysis [33]. Urinary concentrations of t,t-MA and BMA in the KoNEHS were measured using high-performance liquid chromatography–mass spectrometry (HPLC–MS/MS) equipped with Electron Spray Ionization (ESI). This method involves injecting the sample into HPLC–MS/MS after concentrating and purifying it using solid-phase extraction, to analyze t,t-MA and BMA. The value of the sample concentration is determined by reading a calibration curve created using the Standard Addition Method (The determination coefficient (R2) of the curve was ≥0.995.) [35].

Chromatographic separation was performed on a C18 column (3.5 μm; 2.1 × 100 mm) by gradient elution using 0.1% acetic acid solution (purified water) and 0.1% acetic acid solution (methanol) (95:5, *v*/*v*) with a flow rate of 0.3 mL/min. The injection volume was 5 μL and multiple-reaction monitoring (MRM) mode was performed for quantitative analysis. The method detection limits (MDL) for t,t-MA and BMA were 2.3 and 0.197 μg/L, respectively [35].

We utilized a covariate-adjusted standardization (CAS) to account for the effect of urinary dilution. Urinary creatinine (Ucr) levels for each subject were predicted using a linear regression model, incorporating log-transformed Ucr along with significant covariates including age, sex, and BMI. We then calculated the predicted Ucr from this model and obtained the adjusted urinary t,t-MA and BMA levels by dividing the measured urinary t,t-MA and BMA concentrations by the ratio of the measured to predicted Ucr [36].

### 2.5. Statistical Analysis

According to the guidelines of the KoNEHS, all statistical analyses (except restricted cubic smoothing spline regression analysis) were performed using survey procedures. Considering their right-skewed distribution of urinary concentrations of t,t-MA or BMA, natural log-transformed values were employed for statistical analysis. 

Differences in baseline characteristics according to quartiles of the TG/HDL-C ratio were determined using ANOVA tests for continuous variables and Rao–Scott chi-square tests for categorical variables. Associations between t,t-MA and BMA and the suspected cardiovascular disease were assessed using multivariate logistic regression models, estimating ORs and 95% CIs. The urinary concentrations of t,t-MA and BMA were classified into quartiles, with the first quartile as the reference group. Three separate models were applied: the first model was unadjusted; the second model was adjusted for age (continuous variable), sex (in the overall dataset), BMI (continuous variable), smoking status, menopausal status (for women); the third model was further adjusted for the natural log-transformed t,t-MA, or BMA concentration (continuous variable). P for trend was produced by linear regression through regressing the odds ratios.

We also investigated whether there are nonlinear associations, by using restricted cubic smoothing splines with five knots at the 10th, 25th, 50th, 75th, and 90th percentiles. For tests for nonlinearity, we used the likelihood ratio test comparing two models: the model with only the linear term and the model with the linear and the cubic spline terms.

All analyses were performed using the R program (R Foundation for Statistical Computing, Vienna, Austria), and a significance level *p* < 0.05 was considered significant for all analyses. All statistical tests were two-sided, and *p* values < 0.05 were considered significant. 

## 3. Results

The demographic characteristics of the 1928 participants are shown in Table 1. The TG/HDL-C ratio was categorized as the four quartiles. Age, sex, BMI, smoking status, menopausal status (for women), and urinary t,t-MA concentrations in each of the four quartiles are not homogenous (*p* < 0.001). Concentrations of urinary t,t-MA were higher in participants with the higher quartile of the TG/HDL-C ratio. In addition, men, older participants, obese participants, past and current smokers, and postmenopausal women tend to be associated with a higher TG/HDL-C ratio. 

The distributions of t,t-MA and BMA are presented in Table 2, and both are estimated to have right-skewed distributions. The geometric means (GM) of urinary t,t-MA concentrations were 63.66 [59.39, 68.24] in men and 46.81 [44.06, 49.73] in women. The GM of urinary BMA concentrations were 4.15 [3.87, 4.46] in men and 3.81 [3.57, 4.06] in women. The urinary t,t-MA and BMA concentrations were relatively higher in men than in women.

**Figure 2:**
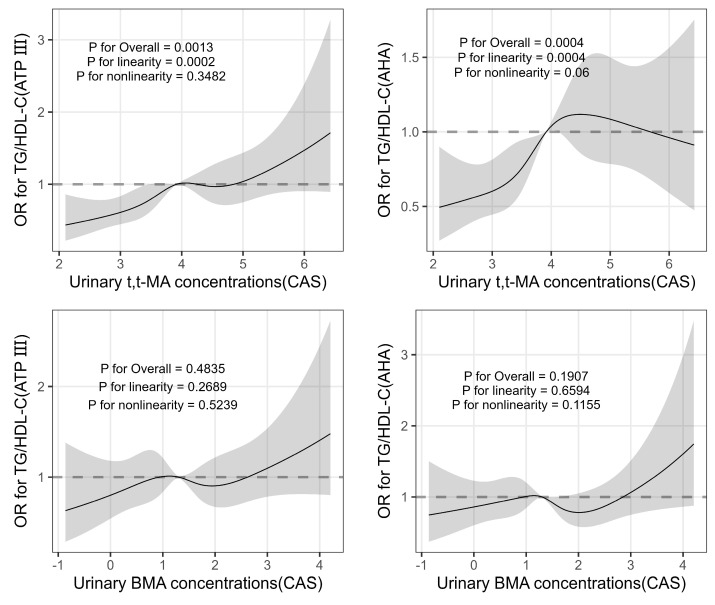
Nonlinear associations between urinary concentrations of t,t-MA and BMA and the TG/HDL-C ratio. A restricted cubic smoothing spline model was used for urinary concentrations of t,t-MA and BMA to allow nonlinear associations. ORs were adjusted for age, sex, BMI, smoking status, menopausal status (for women), and the natural log-transformed t,t-MA or BMA concentration. The dashed lines indicate 95% CIs.

When nonlinear associations between urinary concentrations of t,t-MA and BMA and the TG/HDL-C ratio were evaluated with a restricted cubic smoothing spline function, there were no significant nonlinear associations (Figure 2).

Associations between urinary concentrations of t,t-MA and BMA and TG/HDL-C ratio are given in Table 3. In the continuous model, the risk of an elevated TG/HDL-C ratio was significantly increased per unit of urinary t,t-MA concentrations. This association was prominent in men (OR [95% (CI)]: 1.46 [1.11, 1.91] for ATP III, 1.35 [0.93, 1.95] for AHA). In addition, when survey logistic regression models with categorized variables were conducted, the ORs [95% (CI)] in the highest quartile of the t,t-MA concentrations, compared with the reference quartile, were 1.95 [1.25, 3.05] for ATP III and 1.98 [1.19, 3.27] for AHA, with significant dose–response relationships (P for trend: for ATP III, 0.008, for AHA, 0.006). Furthermore, after stratification with sex, significant associations between urinary t,t-MA concentrations and elevated TG/HDL-C ratio remained in both men and women, with significant dose–response relationships (P for trend: for men, 0.029; women, 0.024). 

On the other hand, urinary BMA concentrations were not associated with an elevation in the TG/HDL-C ratio in both men and women.

## 4. Discussion

This study presents that environmental exposure to benzene is associated with an elevated TG/HDL-C ratio, a surrogate marker for cardiovascular disease in the general population. To our knowledge, only a few studies have assessed the association between environmental exposure to benzene and serum lipid profiles [32]. In this study, significant associations were found between urinary t,t-MA, a metabolite of benzene and elevated TG/HDL-C ratio, and these associations remained in both men and women, after stratification with sex. 

Previous studies have reported the effect of benzene exposure on an elevated TG/HDL-C ratio, insulin resistance, and CVD, which are in line with our results. A cross-sectional study conducted in Louisville, Kentucky, observed an association between benzene exposure and the risk of CVD [37]. The study revealed a positive association between elevated levels of benzene exposure, quantified by t,t-MA, and an augmented susceptibility to CVD among the population. In addition, a study from the Korean Elderly Environmental Panel (KEEP) showed an association between benzene exposure and insulin resistance in the elderly population. The study found that the higher quartiles of urinary t,t-MA were associated with elevated insulin resistance [21]. Another cross-sectional study for children and adolescents also found a significant association between insulin resistance, fasting glucose, and fasting blood insulin with urinary t,t-MA [22]. A recent study of 3423 adults who participated in the Korean National Environmental Health Survey Cycle 3 (KoNEHS, 2015–2017) reported that the TG/HDL-C ratio was elevated as urinary t,t-MA was increased [32]. These results provided evidence that exposure to benzene can have negative health effects.

Globally, large-scale biomonitoring surveys at the national level such as the National Health and Nutrition Examination Survey (NHANES), the Canadian Health Measures Survey (CHMS), and the German Environmental Survey (GerES) provide information on exposure to environmental pollutants in the general population using biomarkers. In NHANES 2017–2018, the GM of urinary t,t-MA in the US population aged 20+ years (1422 individuals) was 55.8 µg/L [48.9, 63.7] [38]. According to the latest report from the CHMS, the GM of urinary t,t-MA for the Canadian population (2514 individuals) was measured at 67 µg/L [61, 74] during Cycle 4 (2014–2015) [39]. In KoNEHS Cycle 3 (2015–2017), the GM of urinary t,t-MA for the Korean adult population (3333 individuals) was reported to be 100 µg/L [94.4, 106]. The concentration of urinary t,t-MA in KoNEHS Cycle 3 exhibited relatively higher levels compared to the United States and Canada [40]. However, the concentration of urinary t,t-MA in current study (53.2 µg/L [50.8, 55.71], KoNEHS Cycle 4 (2018–2020)), was much lower than in the previous cycle, which means that even low-dose exposure to benzene might have a negative effect on cardiovascular health.

The current exposure standards for benzene are established exclusively for workers and do not apply to the general population. Biological Exposure Indices (BEIs), which serve as guidance values representing the concentration of chemicals in the body, play an important means for assessing exposure and health risks to workers. Both the World Health Organization (WHO) and the American Conference of Governmental Industrial Hygienists (ACGIH) established 500 μg/g creatinine as the BEI for t,t-MA. According to the Korea Occupational Safety and Health Agency (KOSHA), biological indicators are categorized as primary, secondary, and recommended categories. Primary indicator substances are essential for health diagnosis and must be conducted as part of the basic examination, and secondary indicator substances are performed when deemed necessary during secondary examinations. KOSHA recommends the reclassification of benzene, presently classified as a secondary indicator, to a primary indicator, in response to the health hazards associated with exposure [41]. 

The primary exposure sources of benzene in the general population without occupational exposure are cigarette smoke [42,43,44], ambient air pollution, and dietary intake [45]. The inhalation of vehicle exhaust and the consumption of canned or pickled food potentially may elevate the levels of urinary t,t-MA in individuals who do not have occupational exposure to benzene [45]. Considering the potential adverse health implications of low-level exposure to benzene, it is imperative to establish exposure thresholds for the general population and implement endeavors aimed at mitigating exposure levels.

Previous study suggested that the generation of reactive oxygen species (ROS), such as hydroxyl radicals and superoxide anions, and the persistent elevation in oxidative stress during benzene metabolism are significant contributors to the toxic effects of benzene [26]. Oxidative stress caused by ROS can lead to oxidative damage to DNA or proteins and lipid peroxidation, which are potential pathophysiological contributors to several diseases like diabetes, hypertension, and CVD [27,28,29,30,31]. Moreover, oxidative stress has been implicated in the pathogenesis of insulin resistance through disruption of insulin signaling and dysregulation of adipocytokines [46,47]. Insulin resistance at the adipocyte results in hormone-sensitive lipase activity, which stimulates the decomposition of triglycerides and the secretion of free fatty acid (FFA). Increased FFA flux to the liver promotes the synthesis of very-low-density lipoproteins (VLDL), resulting in atherosclerosis and hyperlipidemia. In addition, the status of insulin resistance increases Cholesterol Ester Transfer Protein (CETP) activation, which stimulates the exchange process of transferring triglycerides from VLDL to HDL (high-density lipoprotein), LDL (low-density lipoprotein) and receiving cholesterol esters from HDL, LDL. Apolipoprotein A1 (ApoA1) of TG-enriched HDL is well separated from the HDL and excreted in urine, thus reducing reverse cholesterol transport. TG-enriched LDL undergoes lipolysis and turns into small, dense LDL. A decrease in HDL concentration and an increase in small, dense LDL are each risk factors for cardiovascular disease [48].

Although this study shows that environmental low-dose exposure to benzene in the general population might have a negative effect on cardiovascular health, there are several limitations. First, this study was designed as a cross-sectional design of the KoNEHS, which means that the results only indicate associations and do not allow the determination of causal relationships. Therefore, the interpretation should be taken with caution, and the findings of this study need to be replicated through prospective study design. Second, due to the inherent limitations of using secondary data, we were unable to account for other risk factors such as family history of cardiovascular disease, diet, occupation, or air pollution, which could be potential confounders or mediators. Industrial workers in petroleum-, steel-, and chemical-related occupations are constantly exposed to benzene and toluene [49], but data on job codes were not available in KoNEHS. Third, the effects of co-exposure of environmental toxicants on an elevated TG/HDL-C ratio were not investigated. Recently, previous studies proposed that environmental chemical mixtures might be associated with risk of noncommunicable diseases [50]. Therefore, further prospective cohort studies are needed to validate our findings and establish a causal relationship between co-exposure of environmental toxicants and cardiovascular health.

## 5. Conclusions

In conclusion, we found that environmental exposure to benzene was associated with the TG/HDL-C ratio in the general population of Korea. Furthermore, the association persisted across sex, emphasizing the effect of benzene exposure on suspected CVD risk. The key finding of this study showed that even low doses of exposure to benzene might cause adverse health effects, including CVD. Given the potential negative health consequences associated with environmental low-level exposure to benzene, it is crucial to establish a reference value applicable to the general population and undertake concerted efforts to mitigate exposure levels.

## Figures and Tables

**Table 1:** Characteristics of the participants according to quartiles of the TG/HDL-C ratio.

	Overall (n = 1928)	TG/HDL-C Index				
		Quartile 1 (0.36–1.47) (n = 482)	Quartile 2 (1.48–2.46) (n = 482)	Quartile 3 (2.47–4.4) (n = 482)	Quartile 4 (4.41–39.38) (n = 482)	*p*
Age, mean ± SE, years	47.1 ± 0.6	43.5 ± 0.6	47.8 ± 0.7	48.8 ± 0.6	48.4 ± 0.6	<0.001
Sex						
Men, n (%)	803 (41.6)	109 (22.6)	175 (36.3)	229 (47.5)	290 (60.2)	<0.001
Women, n (%)	1125 (58.4)	373 (77.4)	307 (63.7)	253 (52.5)	192 (39.8)	
BMI, mean ± SE, kg/m^2^	24.5 ± 0.1	22.6 ± 0.1	23.9 ± 0.1	25.2 ± 0.2	26.3 ± 0.2	<0.001
Normal weight, n (%)	677 (35.1)	283 (58.7)	194 (40.2)	129 (26.8)	71 (14.7)	<0.001
Overweight, n (%)	456 (23.7)	102 (21.2)	124 (25.7)	114 (23.7)	116 (24.1)	
Obese, n (%)	795 (41.2)	97 (20.1)	164 (34)	239 (49.6)	295 (61.2)	
Alcohol consumption in the past year						
Nondrinkers, n (%)	505 (26.2)	119 (24.7)	132 (27.4)	128 (26.6)	126 (26.1)	0.40
<1 drink/month, n (%)	322 (16.7)	83 (17.2)	64 (13.3)	98 (20.3)	77 (16)	
1 to 2 drinks/month, n (%)	371 (19.2)	90 (18.7)	104 (21.6)	90 (18.7)	87 (18)	
1 to 2 drinks/week, n (%)	433 (22.5)	114 (23.7)	110 (22.8)	102 (21.2)	107 (22.2)	
≥3 drinks/week, n (%)	207 (10.7)	57 (11.8)	49 (10.2)	46 (9.5)	55 (11.4)	
daily intake (%)	90 (4.7)	19 (3.9)	23 (4.8)	18 (3.7)	30 (6.2)	
Smoking status						
Never smokers, n (%)	1309 (67.9)	391 (81.1)	343 (71.2)	317 (65.8)	258 (53.5)	<0.001
Past smokers, n (%)	311 (16.1)	42 (8.7)	86 (17.8)	85 (17.6)	98 (20.3)	
Current smokers, n (%)	308 (16)	49 (10.2)	53 (11)	80 (16.6)	126 (26.1)	
Menopausal status (for women)						
Premenopausal, n (%)	659 (58.6)	279 (74.8)	179 (58.3)	125 (49.4)	76 (39.6)	<0.001
Postmenopausal, n (%)	466 (41.4)	94 (25.2)	128 (41.7)	128 (50.6)	116 (60.4)	
Urinary t,t-MA concentration, mean ± SE, μg/L	92.3 ± 3	89 ± 6.8	86.8 ± 5.4	90.3 ± 5.3	103.1 ± 6.5	0.003
Urinary BMA concentration, mean ± SE, μg/L	7.5 ± 0.4	6.5 ± 0.5	6.4 ± 0.4	7.7 ± 0.7	9.5 ± 1.4	0.14

TG/HDL-C: triglycerides to high-density lipoprotein cholesterol, BMI: body mass index, SE: standard error, t,t-MA: trans, trans-muconic acid, BMA: benzylmercapturic acid.

**Table 2:** Distribution of urinary concentrations in the study participants stratified by sex.

		GM (95% CI)	Min	10%	25th	Median	75th	90%	Max
t,t-MA (μg/L)									
	Overall	53.2 (50.8, 55.71)	1.63	14.49	26.86	49.40	108.24	208.50	1945.96
	Men	63.66 (59.39, 68.24)	2.62	18.57	31.52	62.64	130.55	232.88	1619.97
	Women	46.81 (44.06, 49.73)	1.63	12.93	23.55	44.44	92.36	177.91	1945.96
BMA (μg/L)									
	Overall	3.95 (3.77, 4.14)	0.10	1.14	2.14	4.01	7.44	13.60	467.15
	Men	4.15 (3.87, 4.46)	0.10	1.22	2.33	4.13	7.24	13.40	279.98
	Women	3.81 (3.57, 4.06)	0.10	1.08	2.02	3.95	7.49	13.80	467.15

GM: geometric means.

**Table 3:** Associations between urinary concentrations of t,t-MA and BMA and TG/HDL-C ratio.

		Overall				Men				Women			
t,t-MA (μg/L)		Cases/total	OR (95% CI) ^a^	OR (95% CI) ^b^	OR (95% CI) ^c^	Cases/total	OR (95% CI) ^a^	OR (95% CI) ^b^	OR (95% CI) ^c^	Cases/total	OR (95% CI) ^a^	OR (95% CI) ^b^	OR (95% CI) ^c^
	ATP III												
	Continuous	696/1928	1.44 (1.22, 1.71)	1.34 (1.10, 1.62)	1.34 (1.11, 1.62)	354/803	1.53 (1.23, 1.89)	1.45 (1.09, 1.93)	1.46 (1.11, 1.91)	342/1125	1.20 (0.96, 1.49)	1.21 (0.97, 1.52)	1.21 (0.97, 1.52)
	Quartile 1	127/482	Reference	Reference	Reference	42/143	Reference	Reference	Reference	85/339	Reference	Reference	Reference
	Quartile 2	179/482	1.63 (1.18, 2.24)	1.51 (1.04, 2.19)	1.51 (1.04, 2.19)	78/179	1.62 (0.93, 2.83)	1.67 (0.92, 3.03)	1.66 (0.91, 3.04)	101/303	1.53 (0.99, 2.35)	1.42 (0.88, 2.28)	1.42 (0.89, 2.27)
	Quartile 3	193/482	1.94 (1.32, 2.86)	1.60 (1.06, 2.42)	1.59 (1.06, 2.40)	102/215	2.05 (1.18, 3.57)	1.90 (0.94, 3.84)	1.89 (0.95, 3.77)	91/267	1.61 (0.96, 2.68)	1.35 (0.84, 2.19)	1.35 (0.83, 2.18)
	Quartile 4	197/482	2.34 (1.57, 3.48)	1.94 (1.23, 3.06)	1.95 (1.25, 3.05)	132/178	2.29 (1.43, 3.66)	2.23 (1.14, 4.33)	2.22 (1.17, 4.21)	65/216	1.72 (0.95, 3.11)	1.77 (0.99, 3.19)	1.79 (1.00, 3.22)
	*p* for trend		<0.001	0.009	0.008		<0.001	0.036	0.029		0.070	0.075	0.072
	AHA												
	Continuous	1073/1928	1.37 (1.14, 1.64)	1.27 (1.03, 1.55)	1.27 (1.03, 1.55)	509/803	1.41 (1.04, 1.90)	1.35 (0.93, 1.95)	1.35 (0.93, 1.95)	564/1125	1.17 (0.94, 1.44)	1.19 (0.96, 1.49)	1.20 (0.96, 1.50)
	Quartile 1	214/482	Reference	Reference	Reference	72/143	Reference	Reference	Reference	142/339	Reference	Reference	Reference
	Quartile 2	267/482	1.61 (1.09, 2.38)	1.50 (0.94, 2.42)	1.51 (0.95, 2.38)	113/179	1.97 (1.03, 3.76)	2.10 (1.04, 4.21)	2.10 (1.04, 4.22)	154/303	1.32 (0.82, 2.11)	1.21 (0.69, 2.13)	1.21 (0.71, 2.06)
	Quartile 3	300/482	2.17 (1.38, 3.40)	1.86 (1.13, 3.06)	1.85 (1.13, 3.03)	146/215	2.21 (1.15, 4.27)	2.13 (0.98, 4.63)	2.13 (0.98, 4.62)	154/267	1.91 (1.07, 3.42)	1.68 (0.95, 2.96)	1.65 (0.94, 2.90)
	Quartile 4	292/482	2.27 (1.45, 3.55)	1.97 (1.18, 3.27)	1.98 (1.19, 3.27)	178/178	2.31 (1.21, 4.41)	2.39 (1.05, 5.47)	2.39 (1.05, 5.45)	114/216	1.65 (0.97, 2.81)	1.75 (1.00, 3.03)	1.78 (1.01, 3.11)
*p* for trend			<0.001	0.006	0.006		<0.001	0.036	0.029		0.028	0.024	0.024
BMA (μg/L)		Cases/total	OR (95% CI) ^a^	OR (95% CI) ^b^	OR (95% CI) ^c^	Cases/total	OR (95% CI) ^a^	OR (95% CI) ^b^	OR (95% CI) ^c^	Cases/total	OR (95% CI) ^a^	OR (95% CI) ^b^	OR (95% CI) ^c^
	ATP III												
	Continuous	696/1928	1.19 (0.99, 1.42)	1.07 (0.88, 1.29)	1.07 (0.89, 1.29)	354/803	1.04 (0.81, 1.33)	1.03 (0.81, 1.32)	1.03 (0.81, 1.32)	342/1125	1.28 (1.01, 1.62)	1.12 (0.86, 1.45)	1.12 (0.87, 1.45)
	Quartile 1	148/482	Reference	Reference	Reference	71/174	Reference	Reference	Reference	77/308	Reference	Reference	Reference
	Quartile 2	176/482	1.30 (0.91, 1.84)	1.21 (0.83, 1.74)	1.21 (0.83, 1.74)	93/210	1.13 (0.67, 1.91)	1.04 (0.61, 1.79)	1.05 (0.61, 1.80)	83/272	1.35 (0.83, 2.20)	1.25 (0.74, 2.12)	1.25 (0.74, 2.13)
	Quartile 3	179/482	1.65 (1.10, 2.46)	1.39 (0.85, 2.26)	1.39 (0.85, 2.26)	94/205	1.38 (0.74, 2.60)	1.09 (0.57, 2.09)	1.09 (0.57, 2.09)	85/277	1.85 (1.16, 2.95)	1.68 (0.96, 2.96)	1.69 (0.96, 2.96)
	Quartile 4	193/482	1.52 (1.05, 2.22)	1.19 (0.79, 1.79)	1.19 (0.79, 1.78)	96/214	1.18 (0.70, 2.00)	1.10 (0.68, 1.80)	1.10 (0.68, 1.79)	97/268	1.75 (1.08, 2.82)	1.29 (0.71, 2.35)	1.30 (0.73, 2.33)
	*p* for trend		0.018	0.357	0.355		0.445	0.677	0.683		0.009	0.249	0.234
	AHA												
	Continuous	1073/1928	1.09 (0.91, 1.30)	0.93 (0.79, 1.11)	0.93 (0.79, 1.10)	509/803	1.04 (0.79, 1.37)	1.03 (0.78, 1.36)	1.02 (0.78, 1.35)	564/1125	1.07 (0.84, 1.35)	0.88 (0.69, 1.11)	0.87 (0.69, 1.10)
	Quartile 1	248/482	Reference	Reference	Reference	103/174	Reference	Reference	Reference	143/308	Reference	Reference	Reference
	Quartile 2	272/482	1.25 (0.88, 1.79)	1.14 (0.80, 1.62)	1.13 (0.79, 1.62)	136/210	1.04 (0.63, 1.69)	0.93 (0.53, 1.62)	0.95 (0.54, 1.66)	136/272	1.34 (0.81, 2.23)	1.31 (0.79, 2.17)	1.31 (0.79, 2.17)
	Quartile 3	274/482	1.42 (1.06, 1.90)	1.17 (0.84, 1.64)	1.17 (0.84, 1.64)	137/205	1.59 (0.88, 2.88)	1.29 (0.69, 2.42)	1.30 (0.70, 2.41)	137/277	1.22 (0.87, 1.72)	1.07 (0.70, 1.64)	1.07 (0.70, 1.63)
	Quartile 4	281/482	1.23 (0.89, 1.69)	0.87 (0.61, 1.25)	0.87 (0.61, 1.23)	133/214	1.03 (0.56, 1.90)	0.95 (0.51, 1.79)	0.95 (0.51, 1.78)	148/268	1.24 (0.82, 1.87)	0.80 (0.51, 1.27)	0.80 (0.51, 1.26)
	*p* for trend		0.181	0.584	0.552		0.663	0.908	0.919		0.355	0.350	0.333

t,t-MA: trans, trans-muconic acid, BMA: benzylmercapturic acid, ATP III: National Cholesterol Education Program Adult Treatment Panel III, AHA: American Heart Association, CAS: covariate-adjusted standardization. ^a^ Not adjusted. ^b^ Adjusted for age, sex (for men and women combined), BMI, smoking status, menopausal status (for women). ^c^ Further adjusted for natural log-transformed t,t-MA or BMA concentration.

## Data Availability

This study utilized data from the Korean National Environmental Health Survey, which is accessible to the public.
